# Impacts of COVID-19 on pediatric patients with congenital heart disease: a small systematic and integrative literature review

**DOI:** 10.1590/1984-0462/2025/43/2025073

**Published:** 2025-12-15

**Authors:** Avelyn Moreira Oliveira, Alexandre D’Annibale Cartøgenes, Luigi Chermont Berni, LaÀse Maria Barbosa Amaral, Rafael Lobato Machado, Rita de Cøssia Silva de Oliveira

**Affiliations:** a Universidade do Estado do Pará, Belém, PA, Brasil.

**Keywords:** COVID-19, Congenital heart disease, Pandemic, Pediatrics, COVID-19, Cardiopatia congênita, Pandemia, Pediatria

## Abstract

**Objective:**

The objective of this study was to compile primary studies to understand the impacts of COVID-19 on pediatric patients with congenital heart disease.

**Data source:**

A systematic review based on the Preferred Reporting Items for Systematic reviews and Meta-Analyses (PRISMA) method, with searches conducted in the PubMed, Latin American and Caribbean Health Sciences Literature (LILACS), and Scientific Electronic Library Online (SciELO) databases. Studies published in the last 5 years, open access, and addressing the research question, “What are the main impacts of COVID-19 on pediatric patients with congenital heart disease?” were included. The risk of bias was assessed using the Newcastle-Ottawa Scale (NOS) and the Joanna Briggs Institute (JBI) tools.

**Data synthesis:**

A total of 377 articles were identified, of which 12 met the inclusion criteria. The NOS tool indicated that two of the eight cohort studies had a risk of bias and lower methodological quality. The JBI tool revealed that three of the four cross-sectional studies had a low risk of bias and good methodological quality. The integrative analysis highlighted three main impacts of COVID-19 on these patients: difficulties in follow-up and treatment, reduced physical activity due to social distancing, and postponement of procedures and surgeries. Infected patients experienced increased complications and hospitalizations, but without a significant change in mortality.

**Conclusions:**

The COVID-19 pandemic significantly affected the health and management of congenital heart disease, leading to clinical complications and worsening follow-up. Further primary and secondary studies are needed to strengthen the evidence and improve patient management.

## RESUMO

**Objetivo:**

Compilar estudos primários para compreender os impactos da COVID-19 em pacientes pediátricos com cardiopatia congênita.

**Fontes de dados:**

Revisão sistemática baseada no método *Preferred Reporting Items for Systematic Reviews and Meta-Analyses* (PRISMA), com busca nas bases United States National Library of Medicine (PubMed), Literatura Latino-Americana e do Caribe em Ciências da Saúde (LILACS) e Scientific Electronic Library Online (SciELO). Foram incluídos estudos dos últimos cinco anos, *open access*, que respondiam à pergunta: “Quais os principais impactos da COVID-19 em pacientes pediátricos com cardiopatia congênita?”. O risco de viés foi analisado pelas ferramentas Newcastle- Ottawa (NOS) e Joanna Briggs Institute (JBI).

**Síntese dos dados:**

Foram identificados 377 artigos, dos quais 12 atenderam aos critérios de inclusão. A ferramenta NOS apontou que dois dos oito estudos de coorte apresentaram risco de viés e menor qualidade metodológica. A JBI revelou que três dos quatro estudos transversais tinham baixo risco de viés e boa qualidade. A análise integrativa destacou três principais impactos da COVID-19 nesses pacientes: dificuldades no acompanhamento e tratamento, redução da atividade física por conta do distanciamento social e adiamento de procedimentos e cirurgias. Pacientes infectados tiveram mais complicações e internações, mas sem aumento significativo na mortalidade.

**Conclusões:**

A pandemia impactou significativamente a saúde e o manejo de cardiopatias congênitas, levando a complicações clínicas e piora no acompanhamento. Estudos adicionais são necessários para fortalecer as evidências e aprimorar o tratamento desses pacientes.

## INTRODUCTION

The COVID-19 pandemic was marked by a global health crisis, with the world population suffering from several deaths and sequelae from the infection, generating a demand for rapid advances in healthcare to contain this emergency scenario since its beginning in 2020.^1^ The disease is caused by SARS-CoV-2 virus, with the clinical presentation being heterogeneous, ranging from mild symptoms, such as fever, cough, and fatigue, to more severe forms, which include respiratory failure that requires intubation, neurological manifestations, cardiac complications, and the cytokine storm, characterized as an exacerbated systemic inflammatory response.^2,3^

Initially, the majority of studies focused on the adult and elderly population, since they had more severe cases of the disease, especially in the presence of comorbidities, such as systemic arterial hypertension, diabetes mellitus, cardiovascular diseases, and chronic lung diseases.^4^ These pre-existing health conditions made these individuals more vulnerable to complications from COVID-19, which highlighted the need for medical and scientific attention to this public.^4^

However, it is essential to note that COVID-19 has not been exclusive to these groups. The pediatric population has also been affected by the virus, especially those with congenital heart disease, a condition that involves structural and functional alterations of the heart, being one of the most common forms of birth defect, with approximately 1% of live births manifesting this condition.^5^ These heart diseases can range from simple conditions, such as an atrial septal defect that closes spontaneously, to complex conditions that require several surgeries for proper correction, such as tetralogy of Fallot or transposition of the great vessels.^6^

Although most children have milder forms of COVID-19 when compared to adults and the elderly, patients with heart disease, such as the aforementioned congenital heart diseases, may be at greater risk of developing complications.^7^ The main reason for this is that SARS-CoV-2 infection is associated with cardiovascular diseases such as myocarditis, heart failure, and thromboembolism, contributing to the increased severity and mortality in cases of infection with this virus in this population.^7,8^

The current literature on the effects of COVID-19 in children with congenital heart disease is still limited,^7^ since there are still few studies with moderate or strong scientific evidence in this area.^7^ Therefore, this systematic review aims to fill this gap by reviewing the available evidence on the impacts of COVID-19 on the health of pediatric patients with congenital heart disease.

## METHOD

The searches were made in the following databases: PubMed, Latin American and Caribbean Health Sciences Literature (LILACS) and Scientific Electronic Library Online (SciELO). The descriptors used are primarily listed in the Descriptors in Health Sciences (DeCS) and Medical Subject Heading Terms (MeSH) dictionaries, and the following search strategy was defined along with the Boolean operators: ((Congenital heart disease) OR (Congenital heart defects)) AND ((Children) OR (Pediatric) OR (Infant)) AND ((Covid-19) OR (SARS- CoV-2)). As for the filters selected on PubMed, “Free full text” and “published in the last 5 years” were used. No search filters were applied in the other databases.

This study is a systematic literature review that followed the recommendations of the Preferred Reporting Items for Systematic Reviews and Meta-Analyses 2020 (PRISMA 2020) and was registered with PROSPERO (CRD42024533918).

Observational studies, original in English and Portuguese, available in the PubMed, SciELO, and LILACS databases in the 5 years before the time of the research on March 2024, and addressing the topic of COVID-19 and congenital heart disease were included. The age group included in the review was the entire pediatric population, considering those under 18 years of age in this context. Subsequently, each study was fully analyzed to answer the research question “What are the main impacts of COVID-19 on pediatric patients with congenital heart disease?”

Initially, the Rayyan tool was used to read the titles and abstracts of the studies found by the search method. Those considered to meet the eligibility criteria were selected for a full reading by three reviewers, who carried out an independent assessment using the same criteria that defined whether the study would be included.

The following studies were excluded: reviews, closed-access papers, letters, editorials, duplicates, and articles that neither fit the researched theme nor belong to the stipulated methodological design.

The Newcastle-Ottawa Scale (NOS) and the Joanna Briggs Institute (JBI) critical appraisal tool were used to analyze the risk of bias for cohort and cross-sectional studies, respectively. The NOS assesses three domains: selection, comparability, and outcome, while the JBI assesses the following eight topics: Were the criteria for inclusion in the sample clearly defined?; Were the study subjects and the setting described in detail?; Was the exposure measured in a valid and reliable way?; Were objective, standard criteria used for measurement of the condition?; Were confounding factors identified?; Were strategies to deal with confounding factors stated?; Were the outcomes measured in a valid and reliable way?; and Was appropriate statistical analysis used? Tables were then made to represent the results of this evaluation using Microsoft Excel 2011 software.

From the selection of studies, a table was created to visualize the results obtained, highlighting the main findings of each study and subsequently describing them in a narrative form and discussing them according to the guiding question. Since this study is a systematic literature review and does not involve data collection with human beings, there was no need to go through the research ethics committee.

## RESULTS

**Figure 1:**
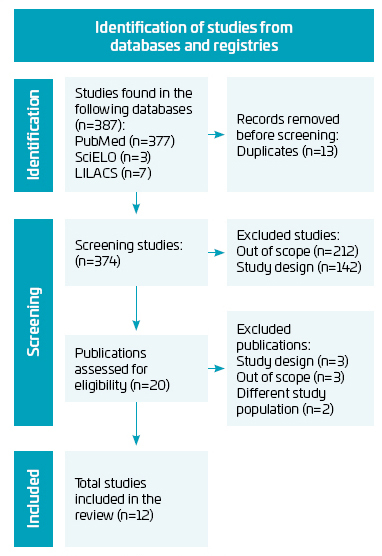
Flowchart of the phases of the systematic review to obtain the articles relevant to the study.

Source: Elaborated by the author, 2025.

From the searches performed in the databases, 377 articles were selected in PubMed, three in SciELO and seven in LILACS, with the application of the stipulated filters, totaling 387 articles. After the exclusion of 13 duplicates, the title and abstract were read, resulting in 20 approved articles, of which 12 studies remained for inclusion in this review after complete reading (Figure 1).

From the 12 studies included, totaling 295,246 patients, there was an investigation of three possible factors that influence the relationship between COVID-19 infection and pediatric patients with congenital heart disease: factors that impacted and modified the continuation of treatment for children with congenital heart disease were mentioned four times, with teleconsultation being one of the ways of continuing treatment; reduced physical activity was another factor mentioned twice, mainly due to social distancing, increasing sedentary lifestyles in this group; the decrease of hospitalizations, procedures and surgeries during the pandemic, mentioned in four articles, since it reduced the risk of exposure of these children to the virus. However, severe cases of COVID-19 in pediatric cardiac patients were rare, but always associated with greater complications and hospitalizations. As for mortality rates, there were no significant changes during this period, except in two articles.

Regarding the methodological designs used, the retrospective cohort study type predominated over the others, totaling five studies out of the 12 selected. The exceptions include: Munaf et al.,^9^ Hemphill et al.,^10^ prospective cohort studies assessing pulmonary changes and modifications in physical activity respectively; Shi et al.,^11^ a multicenter observational cohort study that looked at the impact of COVID-19 on the cardiac surgery program in China; Moghadam et al.,^12^ a cross-sectional study that analyzed COVID-19 infection in children undergoing surgical repairs; Zareef et al.,^13^ a cross-sectional study that analyzed the association between the severity of the virus infection and its complications in pediatric congenital heart disease patients; Honicky et al.,^14^ a cross-sectional study that compared lifestyle changes in children and adolescents with congenital heart disease during the pandemic; Ghimire et al.,^15^ a cross-sectional study that investigated the risk of mortality and cardiovascular complications in pediatric patients with congenital heart disease. Table 1 shows the main characteristics of the studies.

**Table 1:** Characterization of the included studies.

Study	Objectives	Main results
Moghadam et al.^12^	To investigate the status of COVID-19 infection among children who have undergone surgical repair of congenital heart disease in the last 2 years.	A total of 210 patients with CHD were analyzed (mean age 21.6 months; 59.5% female). COVID-19 prevalence was significantly higher than in the general population (p=0.00012), though severe cases were rare, with few ICU admissions and one death. No association was found between ARB/ ACE inhibitor use and infection risk.
Strah et al.^16^	To compare COVID-19 hospitalization outcomes between patients with moderate or severe congenital heart disease and those without CHD.	From April 2020 to March 2021, children and adults with CHD hospitalized for COVID-19 had worse outcomes, longer stays, higher complications, and costs, especially in pediatric cases.
Zareef et al.^13^	To explore the disease course, severity and complications of COVID-19 in patients with concomitant congenital heart disease.	The study included 238 patients, 47.9% with suspected or confirmed SARS- CoV-2, mostly with mild disease. Symptoms were varied, the most common being rhinorrhea and cough (15.6% each) and low fever (11.2%). Only 3.5% required hospitalization, 1.5% ICU care, and one death was reported.
Sachdeva et al.^18^	To assess the impact of COVID-19 on pediatric cardiac care and the role of teleconsultations in ensuring healthcare delivery.	Hospitalizations dropped by two-thirds compared to 2019 (66 vs. 189). Procedures decreased markedly: 84% in catheter interventions, 88% in total CHD surgeries, and 40% in emergency surgeries. Only 15% of 2019 in-person patients (1079 vs. 7176) accessed teleconsultations.
Honicky et al.^14^	To evaluate lifestyle changes in children and adolescents with congenital heart disease during COVID-19 and their association with disease complexity.	During the pandemic, 83.5% reduced physical activity, 37.0% increased sedentary behavior, 26.0% slept more, and 23.6% had poorer diet quality.Overall, 41.8% showed at least one negative lifestyle change. Complex CHD was linked to higher sedentary risk (OR 3.49, 95%CI 1.23–!9.90).
Joshi et al.^21^	Observe the mortality rate and postoperative outcomes of children in the convalescing from COVID-19 undergoing cardiac surgery.	The study analyzed 1129 patients, divided into convalescent (n=349) and control (n=780). Convalescent patients showed no unfavorable outcomes after congenital heart surgery. There was no increased risk of mortality, ICU stay, ventilation time, complications, or serious morbidities.
Munaf et al.^9^	To assess lung changes and clinical outcomes in post-COVID-19 children after congenital heart surgery.	Recovered COVID-19 children needing hospital care had worse lung function, more infections, longer ICU stays, and 20% mortality; home-care patients showed no differences from uninfected children.
Ghimire et al.^15^	To examine the risks of mortality and in-hospital cardiovascular and noncardiovascular complications in pediatric patients with congenital heart disease.	Among 36,690 children hospitalized for COVID-19 in 2020, 3.4% had CHD. CHD did not significantly increase mortality (1.2 vs. 0.8%, p=0.50), but was associated with higher risks of arrhythmias, heart block, respiratory failure, ventilation, and acute kidney injury. The median hospital stay was longer for children with CHD (5 vs. 3 days, p<0.001).
Ehwerhemuepha et al.^23^	To assess the association between previous or pre-existing cardiovascular conditions and the severity of COVID-19 in pediatric patients.	The study analyzed severe COVID-19 — defined as need for oxygen or death — using mixed-effects logistic regression to assess 26 cardiovascular conditions, with multiple comparisons adjusted via the Benjamini- Hochberg method.
Choubey et al.^20^	To study the impact of the COVID-19 pandemic on the treatment of children with heart disease in India in terms of number of outpatient visits, hospitalizations, catheter-based interventions and cardiac surgeries.	In 2020, pediatric cardiac centers saw large reductions in outpatient visits (-74.5%), hospitalizations (-66.8%), surgeries (-73.0%) and catheterizations (-74.3%) compared to 2019. Reductions were smaller for neonates and emergency surgeries. Overall and postoperative mortality increased (8.1 vs. 4.8 and 9.1 vs. 4.3%, respectively).
Hemphill et al.^10^	Quantifying the change in physical activity observed during the initial phase of the COVID-19 pandemic in children with congenital heart disease	During COVID-19, children with CHD showed a 21–24% decrease in weekday physical activity (Fitbit step counts) compared to 2019, while weekends were less affected. Average daily steps remained below the recommended 12,000, with boys more active than girls.
Shi et al.^11^	To evaluate COVID-19’s impact on China’s pediatric congenital heart surgery program and patient outcomes during the outbreak.	Early 2020 saw fewer surgeries, increased emergency cases, and effective use of telemedicine, with no rise in mortality or readmissions.

Source: Elaborated by the author, 2025.

CHD: congenital heart disease; ICU: intensive care unit; OR: odds ratio; CI: confidence interval; ARB: angiotensin receptor blocker; ACE inhibitor: angiotensin-converting enzyme inhibitor.

According to the NOS tool, of the eight studies presented, there were two with risk of bias, since the total score of these studies was below 7, which leads to a reduction in the confidence of the data shown (Table 2). The main area of the scale that was not scored by the articles was the selection criteria, which presupposes difficulties in keeping the exposed group with a good representation.

**Table 2:** Risk of bias for cohort studies using the Newcastle-Ottawa Scale.

Study	Study design	Selection	Comparability	Outcome	Total score
Strah et al.^16^	Retrospective cohort	3	0	3	6/9
Sachdeva et al.^18^	Retrospective cohort	2	0	3	5/9
Joshi et al.^21^	Retrospective cohort	3	1	3	7/9
Munaf et al.^9^	Prospective cohort	3	1	3	7/9
Ehwerhemuepha et al.^23^	Retrospective cohort	4	1	3	8/9
Choubey et al.^20^	Retrospective cohort	4	1	3	8/9
Hemphill et al.^10^	Prospective cohort	4	1	3	8/9
Shi et al.^11^	Observational multicenter cohort	3	1	3	7/9

Source: Elaborated by the author, 2025.

In addition, of the four studies assessed according to the JBI, three had a low risk of bias and good methodological quality, but one point in common was the high risk of bias in domain

5 of the scale, which assesses the identification of possible confounding factors in the choice of groups of people to be included in the study (Table 3).

**Table 3.** Risk of bias for cross-sectional studies using the Joanna Briggs Institute Scale.

Study	D1	D2	D3	D4	D5	D6	D7	D8	Overall
Moghadam et al.^12^	Low	Low	Some concerns	Low	High	Some concerns	Low	Low	Low
Zareef et al.^13^	Low	Some concerns	Some concerns	Some concerns	High	Some concerns	Low	Low	Some concerns
Honicky et al.^14^	Low	Some concerns	Some concerns	Low	High	Some concerns	Low	Low	Low
Ghimire et al.^15^	Low	Low	Low	Low	Low	Low	Low	Low	Low

Source: Elaborated by the author, 2025.

Evaluated domains: D1: Were the criteria for inclusion in the sample clearly defined?; D2: Were the study subjects and the setting described in detail?; D3:Was the exposure measured in a valid and reliable way?; D4:Were objective, standard criteria used for measurement of the condition?; D5:Were confounding factors identified?; D6:Were strategies to deal with confounding factors stated?; D7:Were the outcomes measured in a valid and reliable way?; D8: Was appropriate statistical analysis used?

## DISCUSSION

The COVID-19 pandemic has had a major impact globally, especially on pediatric patients with congenital heart disease. In this context, several studies have investigated the effects of the pandemic on the health and management of these patients, showing significant changes in clinical outcomes, standards of care, and hospital costs.

The study conducted by Strah et al.^16^ analyzed and compared admission rates and length of hospital stay for children with heart disease, showing a significant increase in complication rates and length of hospital stay, which also resulted in higher hospital costs. Other authors corroborate this relationship, such as Ghimire et al.^15^ who showed a prolongation of hospitalizations and the appearance of possible complications, such as tachyarrhythmias and heart blocks, as well as Sanna et al.^17^ who noted more severe forms of COVID-19 in neonates and children with congenital heart disease, with higher rates of intensive care unit admissions and intubations. In addition, Zareef et al.^13^ compiled the main symptoms presented by pediatric patients with congenital heart disease infected by the virus, highlighting rhinorrhea and cough as the most common, while chills were reported less frequently.

On the other hand, Sachdeva et al.^18^ observed a significant reduction in the number of hospitalizations and surgical and nonsurgical procedures during the pandemic when compared to 2019. This reduction is largely due to the social distancing measures and health barriers implemented to contain the spread of the virus. In this scenario, remote support, mainly through teleconsultations, has taken on a leading role in maintaining the healthcare of these patients. A similar observation was made by Shi et al.,^11^ who showed the effectiveness of remote monitoring in reducing hospital visits in less complex cases.

Miana et al.^19^ found an abrupt reduction in surgical volume, accompanied by an increase in the complexity of procedures, which was analyzed at the Hospital das Clínicas and Instituto do Coração in São Paulo, Brazil. However, Choubey et al.^20^ identified that the decrease in hospitalizations was more pronounced in babies and children, while neonates showed only a slight reduction.

In addition to changes in care patterns, behavioral and lifestyle changes during the pandemic have also had a significant impact on the health of children with congenital heart disease. Honicky et al.^14^ described a general worsening in lifestyle habits, including a reduction in physical activity, a worsening in diet quality, and an increase in sleeping hours, often associated with a sedentary lifestyle. These factors can exacerbate complications in congenital heart disease patients. In line with this, Hemphill et al.^10^ quantified the reduction in physical activity using monitoring devices, recording a 21–24% drop in the average number of daily steps among children with heart disease compared to 2019, impact that was more pronounced among girls.

Concerning mortality rates, divergent results have been presented. Joshi et al.^21^ found that, during the pandemic, there was no significant increase in unfavorable outcomes, such as the need for ventilatory support, reintubation, or admissions to intensive care units. Moghadam et al.^12^ also observed a higher prevalence of COVID-19 in pediatric cardiac patients, but no statistically significant change in the severity of cases or the number of complications. In addition, the use of angiotensin receptor blockers and angiotensin-converting enzyme inhibitors, which are frequently prescribed for these patients, was not associated with a higher risk of infection by the vírus.^12^ Corroborating these findings, Sabatino et al.^22^ observed in their cohort study that the pediatric age group had few cardiovascular comorbidities, leading to a mild clinical course of COVID, without mainly modifying mortality.

Contrasting these findings, Ehwerhemuepha et al.^23^ reported an increase in the severity of COVID-19 among pediatric patients with pre-existing pathologies, resulting in higher mortality in children under 12, mainly due to cardiac arrests. Similar results were reported by Munaf et al.,^9^ who compared three groups of children: those who recovered with home care, children with no history of COVID-19, and those who recovered with hospital care. Among the patients in the latter group, there was a higher incidence of lung infections, some of which required mechanical ventilation, increasing in-hospital mortality.

Therefore, all these factors, combined with changes in medical care and lifestyle habits, have significantly influenced the health of children with congenital heart disease during the COVID- 19 pandemic. However, discrepancies remain between the data presented in different studies, highlighting the need for further research to better understand the impacts of this health crisis on this vulnerable group.

About the type of study, retrospective cohort studies predominated, accounting for five of the 12 studies included. Other approaches, such as prospective cohorts and cross-sectional studies, were also used, addressing different aspects such as lifestyle changes, impact on the cardiac surgery program, and cardiovascular complications. However, secondary studies with a higher level of evidence were scarce, such as systematic reviews and meta-analyses, making it difficult to correlate the findings of this study and showing a need for new studies on this subject to improve the management of these patients.

A relevant limitation of this study concerns the literature search strategy, which included only the PubMed, LILACS, and SciELO databases and restricted selection to open-access articles. This choice aimed to ensure the reproducibility of the research, facilitate full-text access for the authors, and promote transparency in the analysis. However, such restriction may have reduced the number of eligible studies, excluding potentially relevant research published in other databases or available only through restricted access, which could impact the comprehensiveness and representativeness of the findings.

Furthermore, most of the included studies did not stratify outcomes based on the type and severity of congenital heart disease, which is an important limitation, as these variables significantly influence clinical results and limit the generalizability of the findings. Another point not addressed in the reviewed studies is the socioeconomic context of the countries and the demographic characteristics of the populations studied. This omission is particularly relevant in the context of telemedicine — often proposed as an alternative to in-person care — which may not be feasible in resource-limited settings, leaving vulnerable patients without adequate access to healthcare services.

Although the reviewed literature indicates that mortality did not increase during the pandemic, a backlog of surgical procedures and an associated trend toward greater clinical severity were observed. This issue deserves attention, as it may have implications for long-term outcomes. Additionally, while the literature mentions increased clinical severity in children with pre-existing comorbidities, it was not possible to clearly identify which specific conditions or congenital heart lesions were most associated with worse outcomes, limiting the ability to perform precise risk stratification.

Finally, the evidence base used is predominantly composed of studies with low methodological quality, including narrative reviews and meta-analyses. This limitation requires caution when interpreting the results and reinforces the need for more robust primary research on this topic.

In conclusion, the COVID-19 pandemic significantly affected pediatric patients with congenital heart disease, leading to changes in clinical outcomes, care standards, and lifestyle habits. Common findings included the use of teleconsultations for continuity of follow-up, reduced physical activity due to isolation, and fewer hospitalizations and elective procedures to limit unnecessary exposure. Severe COVID-19 cases were rare but, when present, were linked to greater complications and prolonged hospitalization. Lifestyle impacts included increased sedentary behavior, reduced physical activity, and poorer diet quality, which may exacerbate heart disease complications. Mortality remained stable in most studies, with few exceptions.

Most evidence came from retrospective cohorts, highlighting the scarcity of higher-level studies and the need for further research. This work, limited to open-access articles, reinforces the importance of generating robust evidence to guide medical management and improve outcomes for this vulnerable group.

## Data Availability

The database that originated the article is available with the corresponding author.
